# Success of Coronal Pulpotomy in Permanent Teeth with Irreversible Pulpitis: An Evidence-based Review

**DOI:** 10.7759/cureus.6747

**Published:** 2020-01-23

**Authors:** Durre Sadaf

**Affiliations:** 1 Conservative Dentistry, College of Dentistry, Qassim University, Buraydah, SAU

**Keywords:** permanent pulpotomy, pulpitis, root canal therapy, bioactive material.

## Abstract

Vital pulp therapies have been used in primary teeth and immature permanent teeth. However, with the advent of new bioactive material, the paradigm is shifting toward permanent teeth with mature apices of roots. There are many prospective and retrospective studies, randomized controlled trials, and systematic reviews that report coronal pulpotomy with bioactive material in permanent teeth with pulpal pathosis proved to be as successful as root canal therapy (RCT). Coronal pulpotomy is cost-effective, not very technical demanding like root canal therapy and less time consuming for both the dentists and patients. This treatment can be offered to the patient as an alternative to endodontic therapy.

The objective of this study is to review the literature related to the clinical outcome of coronal pulpotomy in permanent teeth with mature apex and having pulpal pathosis. This evidence-based review will facilitate clinical decision making in situations to choose coronal pulpotomy over root canal therapy in mature permanent teeth with irreversible pulpitis.

## Introduction and background

A pulpotomy is defined as “the removal of the coronal portion of the vital pulp as a mean of preserving the vitality of the remaining radicular portion” [1]. Coronal pulpotomy is the treatment indicated in immature teeth with pulpal inflammation. The primary objective of pulpotomy is to preserve radicular pulpal tissues that may help to complete apexogenesis in immature permanent teeth. According to American Academy of Pediatric Dentistry (AAPD) guidelines “A pulpotomy is performed in a tooth with extensive caries without evidence of radicular pathology when caries removal results in a carious or mechanical pulp exposure” [2].

Root canal therapy (RCT) is indicated in permanent mature teeth with pulpal and peri-radicular diseases. It involves complete removal of pulp tissues from the root canals, disinfections and restoring them with bioinert materials. Root canal treatment has a high success rate (86.02%) [3]. The survival rate of endodontically treated tooth was reported as 86% at 2-3 years, 93% at 4-5 years, and 87% at 8-10 years [4].

However, root canal treatment is very challenging and technically demanding, as well. A high rate of poor quality obturation (25-62%) and a high percentage of apical periodontitis associated with root-filled teeth (45%) have been observed in various studies [5-7]. Additionally, malpractice claims in endodontics are most commonly associated with technical complications and mishaps [8]. Root canal treatment is also an expensive treatment. A root treated tooth also needs post and core and prosthetic crown that costs additional financial burden for the patients.

With the advent of bioactive materials, a new understanding on pulp regeneration and vascularization, and due to the technical advancement, many researchers are focusing on the coronal pulpotomy in permanent teeth with irreversible pulpal inflammation as another treatment option to root canal therapy [9]. There are a number of studies on coronal pulpotomy showing the success rate comparable to root canal therapy in permanent teeth with pulpal diseases [10-12].

The objective of this study is to review the literature related to the clinical outcome of coronal pulpotomy in permanent teeth with mature apices and with pulpal pathosis. This review will facilitate the clinicians in decision making when treating teeth with pulpal pathosis.

Focused question

A clinical question in the PICO framework was formulated as:

Can complete coronal pulpotomy be an alternate treatment to root canal therapy in patients suffering from pulpal pathosis?

Population (P): Patients with irreversible pulpitis

Intervention (I): Complete coronal pulpotomy

Comparison (C): Root canal therapy

Outcome (O): No signs and symptoms clinically and absence of peri-radicular radiolucency on periapical radiographs.

Search strategy

A well-defined search strategy was developed based on the PICO framework. A combination of appropriate Medical Subject Headings (MeSH) terms and keywords was used. Following databases were searched: PubMed (MEDLINE), EMBASE (OVID), Cochrane Central Register of Controlled Trials (CENTRAL) and Google Scholar. Synonyms were also selected related to irreversible pulpitis, coronal pulpotomy and root canal therapy.

Electronic databases for pulpotomy in permanent teeth with closed apex in irreversible pulpitis until December 2019 were searched. Search terms were “permanent pulpotomy”, “irreversible pulpitis”, “endodontics” and “root canal therapy”. Hand searching from reference list of selected articles, textbooks and endodontic journal was also performed. A total of 461 articles were found. After the removal of duplication and full-text screening, 14 articles were selected for this review.

## Review

In order to achieve a high success rate and to provide high-quality treatment, clinical decisions should be made on the basis of the best available and valid evidence. There have been conflicting opinion about complete pulpotomy in permanent teeth with mature apex with irreversible inflammation of the pulp [13]. The issues associated with coronal pulpotomy in permanent teeth are uncertainty on the pulpal status at the time of treatment, lack of predictability, and absence of any scientific and valid evidence on long term follow-up and success rate [13].

Root canal treatment is considered as a standard of care that produces the most reliable outcome for teeth with pulpal and peri-radicular diseases [14, 15]. The survival rate of root treated teeth is far less than that of vital teeth in spite of excellent treatment outcome with root canal treatment [16]. The most plausible causes may be the lack of proprioceptive mechanism and loss of damping effect [17,18]. Pressoreceptors and proprioceptors are thought to protect the tooth from excessive forces that are lost in root treated teeth. A vital pulp in teeth increases the survival rate of teeth. That is why the vitality of teeth is considered to be beneficial and should be preserved.

An ex vivo study done by Eghbal et al. showed no sign of inflammation on histologic examination of teeth after the direct placement of mineral trioxide aggregate (MTA) on pulpal tissues [19].

Asgary et al. in their multicenter trial compared coronal pulpotomy, accomplished using bioactive material calcium-enriched mixture (CEM), with RCT in permanent teeth with closed apex and irreversible pulpal inflammation. No difference was detected in success rates between pulpotomy and root canal treatment clinically at 6th and 12th months of follow-up, however, radiographically, the pulpotomy group performed significantly better than root canal treatment (P < 0.001) [11].

A high success rate of up to 90% was found in another study done by Alqaderi et al. in which MTA pulpotomy was performed in children in permanent teeth that were indicated for root canal therapy [20].

A case report by Asgary on a molar tooth with irreversible pulpitis with condensing osteitis was treated with coronal pulpotomy using CEM cement. The tooth was followed up for two years. The tooth was clinically asymptomatic, and complete healing of periradicular tissue took place. The calcification of root canals also did not take place; a common phenomenon observed with calcium hydroxide pulpotomy [21].

Taha et al. reported the success rates of 100% in one year and 92.7% at three years in their study with regard to outcome of MTA pulpotomy in mature permanent teeth in which pulp was exposed due to caries [22].

In another study done by Simon et al. pulpotomy with MTA in permanent teeth was found to be highly successful (82% on two years) [23].

In their prospective study on complete coronal pulpotomy using Biodentine in permanent teeth with mature apices and irreversible pulpal inflammation at one year follow-up Taha and Abdulkhader found a high clinical success rate was of up to 100% and radiographic success of up to 93.8% [24].

A study done by Asgary et al. comparing root canal treatment with coronal pulpotomy (CEM pulpotomy) showed no significant difference in success rate between the coronal pulpotomy and root canal treatment over the period of five years [10].

Linsuwanont et al. found a clinical success rate of 87.3% in their study on MTA pulpotomy in cariously exposed pulp in 66 permanent teeth at 62 months follow-up [25].

Asgary et al. compared four types of vital pulp therapies in teeth with irreversible pulpitis in their randomized controlled trial. The success rate of coronal pulpotomy was highest (95.5%) at 12-month follow-up as compared to direct pulp capping (94.7%), and miniature pulpotomy (91.4%) [26].

In a systematic review Aguilar and Linsuwanont compared different types of vital pulp therapies, and, they found MTA pulpotomy with the highest success rate of 96.6% at three years of follow-up [27].

Alqaderi et al. evaluated the success rate of cervical pulpotomy in mature permanent teeth with irreversible pulpitis in their comprehensive systematic review. Overall success rate was reported 94% in one year and 92% in two years. The success rate with bioactive materials, i.e. MTA, was more as compared to calcium hydroxide pulpotomy. Due to the high success rate, the author of this systematic review proposed this treatment (coronal pulpotomy) in permanent teeth with irreversible pulpitis as a viable treatment [28].

Another systematic review by Cushley et al. evaluated the clinical success rate of coronal pulpotomy in permanent teeth presented with symptomatic irreversible pulpitis. Different types of study designs - prospective, retrospective and randomized controlled trials - were included in this systematic review. The success rate of coronal pulpotomy was found 97.4% clinically and radiographically 95.4% at 12-month follow-up [29].

A systematic review by Li et al. reported on comparison between MTA pulpotomy and calcium hydroxide pulpotomy. Pulpotomy with MTA has been found higher clinical and radiographic success rate at 12 months (Odds ratio: 2.23, 95% CI: 1.16-4.29, P = 0.02) as compared to that of calcium hydroxide pulpotomy [30].

Results of all clinical studies and systematic reviews showed a favorable outcome of pulpotomy in mature permanent teeth with irreversible pulpitis at two or three years and five years of follow-up.

Failure of coronal pulpotomy has been observed most commonly in teeth with defective coronal restoration that can cause microleakage [31]. The most crucial factor in the favorable outcome of vital pulp therapy is adequate sealing with bioactive material and coronal restoration [20, 32, 33]. Microleakage through restoration might be the reason for the decrease in survival rate of pulpotomy over time (One-year Weighted success rate: 94%; two-year Weighted mean difference: 92%). It shows that regular evaluation and follow-ups of restoration is critical to ensure marginal integrity and repairing any defective restoration in time [28]. A study showed the highest success rate of pulpotomy with prosthetic crown followed by amalgam and composite showed poor performance among three [33].

Root canal treatment involves a considerable loss of tooth structure. Many studies showed that the most common reason for failure of root canal treatment is fracture due to inadequate remaining tooth structure [17, 34]. Coronal pulpotomy is a more conservative therapy and a significant tooth structure is preserved in pulpotomy.

Recent development in bioactive materials has been found to be very useful in the treatment of irreversibly inflamed pulpal tissues. Most commonly used bioactive materials are calcium silicate-based material (MTA, Biodentine) and CEM. Both types of materials are biocompatible and capable of inducing cementogenesis, dentinogenesis and osteogenesis [35, 36]. MTA and CEM have been found exhibiting better success rates than calcium hydroxide due to biocompatibility and excellent sealing ability [37, 38].

## Conclusions

Many times patients are unable to receive root canal treatment due to the cost and lack of insurance and they have no choice other than extraction of their teeth. Coronal pulpotomy is less invasive, cost-effective, simple and less time-consuming for patients and the dentists. Systematic review and meta-analysis are considered as the highest level of evidence. All systematic reviews on coronal pulpotomy showed a high success rate compared to root canal therapy. Coronal pulpotomy is an evidence-based safe and predictable treatment that can be offered to adult patients in teeth with irreversible pulpitis as a substitute to root canal therapy.
